# Comparison of labetalol and lidocaine in induction of controlled hypotension in tympanoplasty: a randomized clinical trial

**DOI:** 10.1016/j.bjorl.2024.101403

**Published:** 2024-02-22

**Authors:** Ali Karami, Zeinabsadat Fattahi Saravi, Hossein Hosseini, Mehdi Rahmati, Reza Jahangiri, Naeimehossadat Asmarian, Mahsa Banifatemi

**Affiliations:** a Anesthesiology and Critical Care Research Center, Shiraz University of Medical Sciences, Shiraz, Iran; b Student Research Committee, Shiraz University of Medical Sciences, Shiraz, Iran; c Otolaryngology Research Center, Shiraz University of Medical Sciences, Shiraz, Iran

**Keywords:** Tympanoplasty, Labetalol, Lidocaine, Controlled hypotension, Bleeding

## Abstract

**Objectives:**

This study aimed to compare the efficacy of labetalol and lidocaine in tympanoplasty surgery, specifically evaluating their impact on hemodynamic changes and perioperative outcomes.

**Methods:**

A randomized controlled trial was conducted with 64 patients scheduled for tympanoplasty. Patients were randomly assigned to receive either 0.5–2 mg/min labetalol or 1.5 mg/kg/h lidocaine 1% to achieve controlled hypotension during surgery. The efficacy of the drugs was assessed by comparing the Mean Arterial Pressure (MAP), surgeon's satisfaction, time to target MAP, bleeding volume, postoperative pain scores, the need for analgesic medication in recovery, sedation, and other additional parameters.

**Results:**

The hemodynamic parameters showed a similar trend over time in both the labetalol and lidocaine groups. The median bleeding volume in the labetalol group (10 cc) was lower than that in the lidocaine group (30 cc), although this difference was not statistically significant (*p* = 0.11). Similarly, surgeon's satisfaction level, pain intensity, and sedation level in the recovery room did not show statistically significant differences between the two groups (*p* > 0.05). The duration of surgery, recovery stay, and extubation time also did not significantly differ between the groups. Both medications took approximately the same time (20 min) to reach the target MAP and exhibited comparable hemodynamic responses (*p* > 0.05).

**Conclusion:**

Both labetalol and lidocaine effectively achieved controlled hypotension during tympanoplasty surgery, thereby improving surgical conditions. The choice of medication should be based on individual patient characteristics and the anesthesiologist's judgment.

**Level of evidence:**

II.

## Highlights

• Both lidocaine and labetalol effectively achieved the desired hypotension levels.
• Both drugs achieved the target blood pressure within a similar time frame.
• Both drugs have comparable hemodynamic response in achieving hypotension.
• There is a comparable quality of surgical field visualization with both drugs.
• The choice of drug does not impact the overall duration of the surgical procedures.

## Introduction

Controlled hypotension is a critical component of middle ear surgery, as it ensures optimal visualization of the surgical field and minimizes bleeding.^1^, ^2^ This strategy has proven effective in reducing surgical hemorrhage and improving overall surgical outcomes.^3^ While Monitored Anesthesia Care (MAC) is generally safe,^4^ general anesthesia is preferred for complex or lengthy procedures to minimize patient discomfort.^5^ The choice of medication is vital in patients undergoing tympanoplasty to prevent complications related to bleeding and hemodynamic fluctuations.^5^ Although various drugs have been successful in inducing hypotension, the effectiveness of lidocaine infusion in this context remains inconclusive.

Labetalol, a nonselective β-blocker, has demonstrated established efficacy in achieving controlled hypotension across various surgical procedures, including tympanoplasty.^6^ Studies have consistently reported lower heart rates, mean arterial pressure, reduced bleeding, and improved surgical field visibility with labetalol compared to other agents.^7^, ^8^ On the other hand, lidocaine, an intravenous local anesthetic, has the potential to induce hypotension but requires deep general anesthesia.^9^, ^10^ Lidocaine's ability to lower blood pressure is attributed to its negative inotropic effect on the heart and suppression of sympathetic nervous system stimulation.^11^ A clinical trial has highlighted the advantages of lidocaine-induced hypotension, including improved surgical outcomes, shorter extubation time, reduced fentanyl usage, and decreased postoperative pain.^12^ Although lidocaine is not as commonly employed as a β-blocker for inducing hypotension, it presents potential benefits due to its properties such as local anesthesia, reduction of intracranial pressure, and suppression of the cough reflex.^13^ However, the existing literature lacks sufficient evidence on the efficacy of lidocaine infusion in inducing hypotension during tympanoplasty surgery.

Considering these factors, our study aims to compare the effectiveness of labetalol and lidocaine in achieving controlled hypotension during tympanoplasty, with a specific focus on perioperative hemodynamic parameters, surgical characteristics, and potential complications. We hypothesize that patients induced with either drug infusion will not significantly differ in terms of bleeding volume and surgical field visibility during the perioperative period. This study will contribute to the existing knowledge and understanding of medication choices for inducing controlled hypotension in the context of middle ear surgery.

## Methods

### Study overview

This double-blind clinical trial was conducted between March 2019 and November 2020. The study protocol received ethical approval from the ethics committee of Shiraz University of Medical Sciences, Shiraz, Iran (ID: IR.SUMS.MED.REC.1397.391). Additionally, the trial was registered with the Iranian Registry of Clinical Trials in the WHO registry network on March 1, 2019 (trial ID: IRCT20180922041084N3). The study included 64 patients, aged 18–60 years, classified as ASA (American Society of Anesthesiologists) I and II, who were selected from Khalili and Dastgheib Hospitals in Shiraz, Iran. All participants provided written informed consent, and the study was conducted in accordance with the Helsinki Declaration-2013 and CONSORT guidelines.

**Figure 1:**
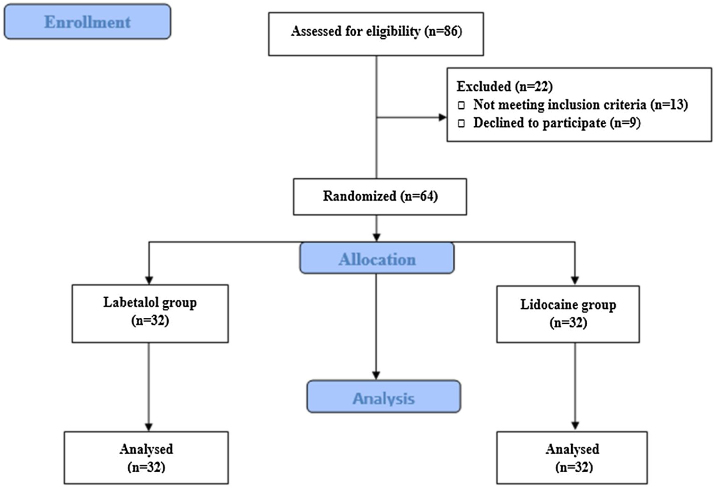
Consort flow diagram.

Initially, 86 patients were assessed for eligibility, and ultimately, 64 patients (32 in the Labetalol group and 32 in the Lidocaine group) completed the study (Fig. 1). The study enrolled patients with subtotal dry perforation and a minimum remnant of the tympanic membrane measuring at least 1 mm. Exclusion criteria included sensitivity to labetalol and lidocaine, history of hypertension and cardiac disorders, liver and kidney diseases, hormonal disorders, chronic pain, gastrointestinal disorders, sexual disorders, brain and respiratory disorders, hematologic disorders, pregnancy, and drug addiction or the use of sedatives or anticoagulants.

### Intervention

All surgical procedures were performed exclusively by a single otologist surgeon using a postauricular approach under general anesthesia. The postauricular approach is conventionally carried out under the administration of general anesthesia.^14^ The standardized induction dose of anesthesia included midazolam (0.03 mg/kg), fentanyl (2 µ/kg), thiopental (5 mg/kg), atracurium (0.6 mg/kg), and morphine (0.15 mg/kg for patients with systolic pressure > 80 mmHg). In the labetalol group, patients received a single intravenous bolus of labetalol (20 mg) over 2 min, followed by an intravenous infusion of labetalol (0.5–2 mg/min) with a maximum dose of 300 mg per patient. In the lidocaine group, patients received an intravenous bolus of lidocaine (1.5 mg/kg, 1%) within 2-min after intubation, followed by a lidocaine infusion at a rate of 1.5 mg/kg/h.

Throughout the entire surgical procedure, the surgeon utilized an otoscopic microscope. Temporalis fascia harvested from the dried temporalis muscle was used as the graft material for myringoplasty. Intraoperative fluid management followed the 4-2-1 rule for maintenance fluid requirement, using 0.9% saline. If a patient's mean arterial pressure dropped below 55 mmHg, a bolus of 5 mg ephedrine was administered. During the procedure, the temporoparietal flap was elevated, and the desiccated temporalis fascia was inserted using the underlay technique. Gelfoam was employed both medially and laterally to secure the graft, followed by wound closure using vicryl sutures. The entire surgical process, from incision to closure, typically lasted about 60 min. Additional time is allocated to pre-surgical preparations and post-procedure wound dressing. During the surgery, if a patient's heart rate exceeded 100 beats per minute or if their blood pressure exceeded to a level greater than 120 percent of baseline, 50 µg of fentanyl was administered. Further, if the patient was resistant to fentanyl, a nitroglycerin bolus of 50–100 µg was given. Also, 5 mg of ephedrine was injected as a bolus if mean arterial pressure decreased to less than 55 mmHg.

In the recovery ward, patients reported a pain score greater than 4 on the Visual Analog Scale (VAS) were given a 1 g diluted dose of Apotel® (Acetaminophen injection 150 mg/mL) over 15 min. Additionally, 50 mg of pethidine was prescribed as needed for pain relief. If a patient's systemic blood pressure in the recovery ward dropped below 80 mmHg, fluid therapy was administered, and the patient's bleeding was monitored. Bradycardia, defined as a heart rate below 45 beats/min, was managed by administering 0.015 mg/kg of atropine intravenously.

### Sample size, randomization, and blinding

The sample size was determined based on a pilot study conducted at the onset of our project, considering an alpha (α) value of 0.05, a test power of 80%, and an effect size of 70%. Sixty-four eligible patients were randomly assigned to either the labetalol group (n = 32) or the lidocaine group (n = 32) using the block randomization method (www.sealedenvelope.com), with a block size of 4. The randomization sequence was generated by a statistician who was not involved in the study and was kept in opaque, sealed envelopes, numbered consecutively. The allocation concealment was maintained until the completion of the study. Both the patients and the surgeon were blinded to the intervention allocation. The labetalol and lidocaine solutions were prepared by a pharmacist who was not involved in the study and were delivered to the operating room in identical syringes labeled with the patient numbers. The anesthesiologist who administered the intervention was not involved in the data collection or analysis. The code was broken, and data were analyzed after the completion of the study.

### Clinical data

The primary outcome measure was the intraoperative Mean Arterial Pressure (MAP) recorded at 5-min intervals during the surgical procedure, as well as the postoperative assessment of bleeding intensity by the surgeon. Secondary outcome measures included Heart Rate (HR) at the same time points, intraoperative bleeding volume, surgical duration, postoperative pain scores, and the need for analgesic medication in the recovery ward. Additional recorded data included Systolic Blood Pressure (SBP), Diastolic Blood Pressure (DBP), arterial oxygen saturation, exhaled carbon dioxide levels, fluid intake, quality of sedation, and postoperative extubation time.

The target MAP was defined as either a mean blood pressure decreases of 30% from the baseline MAP before induction or a MAP within the range of 50–65 mmHg. The early response time referred to the duration it took for a patient's blood pressure to reach the target MAP. The hemodynamic response was evaluated by calculating the ratio of the time a patient maintained the target MAP to the total duration of surgery, expressed as a percentage: (Duration of target MAP)/(Total duration of surgery) ×100.

To assess pain experienced by the patients, the Visual Analogue Scale (VAS) was utilized. The VAS consists of a 10 cm long scale ranging from 0 (indicating “no pain”) to 10 (indicating the most unbearable pain). Sedation levels were evaluated using the Richmond Agitation-Sedation Scale (RASS), which offers ten possible scores ranging from −5 to 0 and then to +4. Negative scores indicate varying degrees of sedation, while positive scores indicate different levels of agitation.

The surgeon's assessment of bleeding intensity in the surgical field was categorized using a 4-item Likert scale, with the following options: “dissatisfied”, “neutral”, “satisfied”, and “completely satisfied”.

### Statistical analysis

Data were analyzed using SPSS 21 (IBM, USA). Continuous variables were reported as mean ± SD or median (IQR), and independent sample *t*-tests and Mann-Whitney *U* tests were used for continuous variables. Categorical variables were reported as numbers and percentages, and the Chi-Square test and Fisher's exact test were used to analyze differences in categorical outcome variables. Repeated measure ANOVA tests were conducted for data obtained over time. A significance level of *p* < 0.05 was used to determine statistical significance.

## Results

A total of 64 patients (22 males and 44 females) received the assigned interventions, with 32 in the labetalol group and 32 in the lidocaine group. All patients underwent tympanoplasty using the postauricular approach with the aid of general anesthesia. Table 1 presents the baseline characteristics of the patients.

**Table 1:** Baseline characteristics of the study patients.

	Labetalol (n = 32)	Lidocaine (n = 32)
Age (year)	37.06 ± 9.05	41.83 ± 11.24
Sex, Male (n, %)	13 (40)	9 (28)
Body weight (Kg)	64.7 ± 11.1	65.1 ± 8.3
ASA (I/II)	25/7	27/5
SBP (mmHg)	125.5 (118.25–138.5)	125 (118.5–136)
DBP (mmHg)	85 (77.5–88.25)	84 (79–91)
MAP (mmHg)	99.5 (94.25–108.25)	96.5 (91.25–110)
HR (beats/min)	88.5 (77.5–97)	82.5 (73.25–92.75)
SpO_2_ (%)	98 (97–100)	99 (98–100)
ETCO2 (mmHg)	32 (30–36.5)	30 (28–33.5)

The values indicate mean ± SD or median (Q_1_‒Q_3_) or number of patients (percentage).

**Figure 2:**
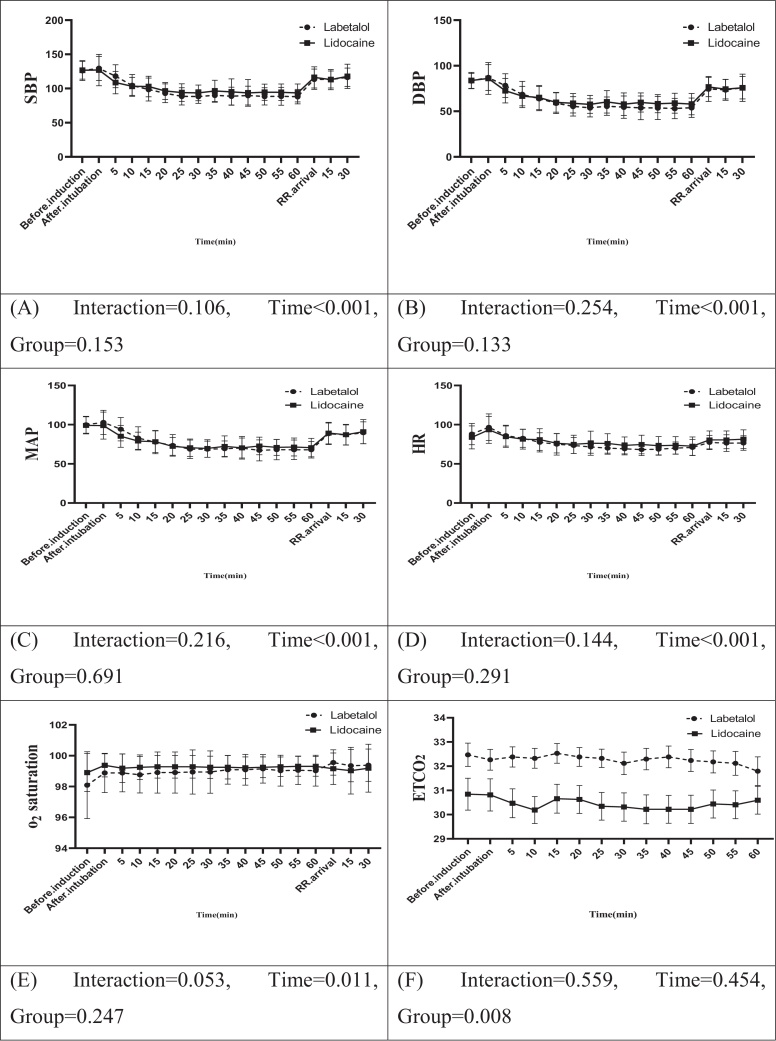
Comparison of repeated measures ANOVA analysis of studied variables in the two groups.

Fig. 2 illustrates a summary of the repeated measures analysis of variance (ANOVA) for hemodynamic factors and certain study variables. The results showed no statistically significant interaction between the two groups throughout the study in terms of SBP, DBP, MAP, HR, oxygen saturation, pain intensity, and sedation level (*p* > 0.05) during both the intraoperative and recovery stages. However, the labetalol group demonstrated significantly higher levels of End-Tidal Carbon Dioxide (ETCO2) compared to the lidocaine group, both in the operating room and the recovery room (*p* = 0.008).

According to Table 2, the median earliest times for patients treated with labetalol and lidocaine to reach the target MAP were 20 min (*p* = 0.83). The hemodynamic response was 58.5% in the labetalol group and 49.5% in the lidocaine group (*p* = 0.22). There were no significant differences between the two drugs in these variables.

**Table 2:** Comparison of earliest response time and percentage of whole response time to the drugs across the groups.

	Labetalol (n = 32)	Lidocaine (n = 32)	*p*-value
Early response time (min)	20 (15–30)	20 (10–40)	0.83
Hemodynamic response (%)	58.5 (37–76)	49.5 (23.5–70)	0.22

“Early response time” refers to the time taken to reach the target mean arterial blood pressure. “Hemodynamic response” indicates the percentage of the time period in which patients-maintained blood pressure within the targeted values during the tympanoplasty surgery.

Based on Table 3, the duration of the operation did not significantly differ between patients who received labetalol and those who received lidocaine (*p* = 0.32). Approximately half of the operations lasted 107.5 min or less in the labetalol group and 100 min or less in the lidocaine group. The median volume of bleeding in the labetalol group was lower than that in the lidocaine group (10 [5–30] vs. 30 [6.25–50] cc), but this difference was not statistically significant (*p* = 0.11). The median volume of blood loss compensated in the lidocaine group was higher than in the labetalol group (100 [0–150] vs. 0 [0–100] cc), but this difference was not statistically significant (*p* = 0.059). The results indicated no significant differences between the two groups in terms of extubation time, recovery stay, ephedrine use, intra- and postoperative fentanyl use, and the first intraoperative and recovery requests for narcotic drugs (*p* > 0.05).

**Table 3:** Comparison of study variables during surgery and in the recovery room between groups.

	Labetalol (n = 32)	Lidocaine (n = 32)	*p*-value
Bleeding volume (cc)	10 (5–30)	30 (6.25–50)	0.11
Bleeding compensation (cc)	0 (0–100)	100 (0–150)	0.05
Tympanoplasty duration (min)	107.5 (80–157.5)	100 (71.25–150)	0.32
Duration of end of operation to extubation (min)	15 (15–25)	20 (15–30)	0.20
Length of stay in recovery room (min)	45 (40–60)	47 (45–60)	0.39
Ephedrine use (mg)	0 (0–10)	0 (0–5)	0.26
Intraoperative fentanyl use (μg)	0 (0‒0)	0 (0‒0)	0.63
Postoperative fentanyl use (μg)	0 (0‒0)	0 (0‒0)	0.68
Time to the first intraoperative call of narcotic (min.)	0 (0‒0)	0 (0‒0)	0.57
Time to the first postoperative call of narcotics (min.)	0 (0‒0)	0 (0‒0)	0.59

The values indicate as median (Q_1_‒Q_3_).

We did not observe any significant difference in the subjective assessment of the surgical field between the use of labetalol and lidocaine in the two groups (*p* = 0.33). Only one surgeon expressed dissatisfaction in the lidocaine group, while 65% of surgeons in both the labetalol and lidocaine groups reported satisfaction (Table 4).

**Table 4:** Surgeon's assessment of surgical field visibility.

Surgeon Satisfaction	Labetalol (n = 32)	Lidocaine (n = 32)	*p*-value
Dissatisfied	0 (0)	1 (3.1)	0.33
Neutral	2 (6.25)	5 (15.6)	
Satisfied	21 (65.62)	21 (65.6)	
Completely Satisfied	9 (28.12)	5 (15.6)	

The values indicate frequency (percentage).

Regarding adverse effects, four patients in the labetalol group and two patients in the lidocaine group experienced profound hypotension (MAP < 50 mmHg) for at least 5 min. In one patient from the labetalol group and three patients from the lidocaine group, the mean arterial pressure did not reach the target MAP level. Additionally, three patients in the labetalol group and two patients in the lidocaine group had bleeding volumes greater than 50 cc. Two patients in the labetalol group exhibited an unconscious level of sedation (−5) and a highly agitated state (+3) in the recovery room. One patient in the lidocaine group had moderate sedation (−3) during recovery. None of the patients reported bradycardia (HR < 45) during the study.

## Discussion

This study compared the effectiveness of lidocaine and labetalol in achieving hypotension during middle ear surgery. Both drugs demonstrated similar efficacy in reducing intraoperative hemodynamic parameters associated with induced hypotension, with comparable mean arterial pressure values in both groups. Our study is the first randomized clinical trial to directly compare the effects of lidocaine and labetalol in this specific surgical context, offering valuable insights into their respective roles in achieving controlled hypotension.

The time required to reach the target MAP was similar in both lidocaine and labetalol groups, with a median of 20 min. This finding aligns with a previous study by Kamel et al.,^15^ which reported a comparable time for achieving the target MAP using intravenous magnesium sulfate. However, Kamel et al.^15^ observed a faster time of 6.9 ± 1.5 min for their labetalol group, which is considerably shorter than our labetalol group. It should be noted that their patients received labetalol as an intravenous bolus over 15-min, whereas in our study, it was administered as a 2-min intravenous bolus. Additionally, Kamel et al.^15^ measured the time from the end of the bolus dose until the target MAP was reached, which may explain the discrepancy.

Our study found that the labetalol group exhibited significantly higher levels of End-Tidal Carbon Dioxide (ETCO2) compared to the lidocaine group during intra- and post-operative periods. This finding is consistent with previous research by De Hert et al.,^16^ who reported that intravenous labetalol administration during middle ear surgery leads to statistically significant changes in arterial oxygen (PaO_2_) and carbon dioxide (PaCO_2_) levels, although these changes have only minor clinical implications. Seok Do et al.^17^ also demonstrated that labetalol attenuates the hemodynamic changes caused by tracheal intubation and inspired desflurane.

Labetalol acts as a nonselective β-blocker and α-adrenoceptor antagonist, resulting in reduced blood pressure, decreased reflex tachycardia, and unchanged or increased cardiac output.^6^ It has been extensively studied and proven effective in achieving controlled hypotension during various surgeries.^7^ On the other hand, the exact mechanisms by which lidocaine induces hypotension are not fully understood, but previous research suggests that its negative inotropic effect and suppression of the sympathetic nervous system may play a role.^18^ Lidocaine, as an intravenous local anesthetic, has the potential to induce controlled hypotension,^10^, ^18^ but deep general anesthesia is a prerequisite for its hypotensive effects.^10^ In a clinical trial involving patients undergoing sinus surgery, Omar reported several advantages of lidocaine, including improved visibility during surgery, shorter extubation time, reduced fentanyl use, and less postoperative pain.^12^ However, it should be noted that functional endoscopic sinus surgery and tympanoplasty are different procedures with unique considerations. Sinus surgery is prone to bleeding due to the rich vascular supply of the nasal mucosa. Ear surgery, including tympanoplasty, may yield better outcomes with intravenous lidocaine. In our trial, we observed a potential trend towards improved bleeding control and resuscitation fluid use with labetalol, although these differences were not statistically significant. The subjective visual assessment of the surgical field may be influenced by individual surgeon bias and may not always correlate with objective measures of bleeding volume. Additionally, the ideal visualization of the surgical field for each hypotensive drug occurs at different target MAP levels.^8^

Controlled hypotension has been shown to reduce surgical duration by improving visibility of the surgical field and minimizing time spent on repeated suctioning. In our study, the duration of the operation, recovery stay, and extubation time did not significantly differ between the lidocaine and labetalol groups, suggesting similar surgical and recovery time requirements. Moreover, our results demonstrate that both groups-maintained blood pressure within the target range for approximately half of the surgical duration, indicating comparable hemodynamic responses. However, it is important to note that the success rate of achieving controlled hypotension is a different measure of efficacy. Alkan et al. reported a success rate of over 90% for achieving controlled hypotension in different hypotensive agents, with the nitroglycerin group achieving a 100% success rate.^19^ In comparison, we found that the mean arterial pressure successfully reached the target level in 96.8% of patients in the labetalol group and 90.6% in the lidocaine group. Therefore, it is necessary to consider the targeted blood pressure and the mechanism of action of the drug when comparing different studies.

Our study revealed comparable postoperative analgesic effects between the lidocaine and labetalol groups, as evidenced by similar postoperative pain scores and analgesic requirements. A Cochrane meta-analysis concluded that the impact of intravenous lidocaine on pain scores, opioid consumption, and recovery is uncertain and unlikely to have a clinically relevant effect beyond 24 h.^20^ However, a clinical trial reported potential advantages of lidocaine in terms of reduced postoperative pain and opioid consumption compared to placebo.^12^ It should be noted that pain perception and analgesic requirements can vary among individuals, and further research is needed to fully understand the analgesic effects of lidocaine and labetalol in different surgical contexts.

In terms of safety, both lidocaine and labetalol were well-tolerated in our study, with no significant differences in adverse events between the two groups. Lidocaine is generally considered safe when administered within the recommended dosage range,^11^ although rare adverse events such as central nervous system toxicity and cardiovascular effects can occur.^21^ Labetalol, as a beta-blocker, can potentially cause adverse effects such as bradycardia, bronchospasm, and hypoglycemia, particularly in susceptible individuals.^22^ However, in our study, no severe adverse events were observed in either group. Close monitoring of vital signs and appropriate patient selection are essential to minimize the risk of complications associated with these drugs.

There are several limitations to our study that should be considered. First, the sample size was relatively small, which may limit the generalizability of our findings. Further studies with larger sample sizes are needed to validate our results. Second, our study focused specifically on middle ear surgery, and the findings may not be applicable to other surgical procedures. Different surgeries may have unique hemodynamic requirements and responses to hypotensive agents. Third, our study only evaluated the intraoperative and immediate postoperative periods. Longer-term outcomes and complications were not assessed. Future studies should investigate the effects of lidocaine and labetalol on postoperative outcomes, such as wound healing, infection rates, and long-term patient satisfaction.

## Conclusion

In conclusion, our study demonstrated comparable efficacy between lidocaine and labetalol in achieving controlled hypotension during middle ear surgery. Both drugs effectively reduced intraoperative hemodynamic parameters, with similar mean arterial pressure values and time to reach the target blood pressure range. The choice between lidocaine and labetalol may depend on various factors, including the surgeon's preference, patient characteristics, and the specific surgical context. Further research is warranted to explore the optimal use of these drugs in different surgical settings and to evaluate their impact on postoperative outcomes.

## Trial registration

This study was approved by ethics committee of Shiraz University of Medical Sciences, Shiraz, Iran on June, 2018 (Ethical Committee Approval ID: IR.SUMS.MED.REC.1397.391). This clinical trial was also registered at Iranian Registry of Clinical Trials, one of primary registries in the WHO registry network, at https://www.irct.ir/trial/36403 on 1 March 2019 (trial registration No: IRCT20180922041084N3).

## Conflicts of interest

The authors declare no conflicts of interest.
